# P-710. Genomic Epidemiology and Temporal Dynamics of Respiratory Syncytial Virus in Kyoto, Japan, 2011–2024

**DOI:** 10.1093/ofid/ofaf695.922

**Published:** 2026-01-11

**Authors:** Yasufumi Matsumara, Yusuke Tsuda, Koh Shinohara, Yasuhiro Tsuchido, Masaki Yamamoto, Miki Nagao

**Affiliations:** Kyoto University Graduate School of Medicine, Kyoto, Kyoto, Japan; Kyoto University Graduate School of Medicine, Kyoto, Kyoto, Japan; Kyoto University Graduate School of Medicine, Kyoto, Kyoto, Japan; Kyoto University Graduate School of Medicine, Kyoto, Kyoto, Japan; Kyoto University Graduate School of Medicine, Kyoto, Kyoto, Japan; Kyoto University Graduate School of Medicine, Kyoto, Kyoto, Japan

## Abstract

**Background:**

Respiratory syncytial virus (RSV) is a major cause of acute lower respiratory tract infections in children, but recent data indicate a significant disease burden in adults as well. In Japan, limited knowledge of the clinical and molecular epidemiology of RSV is available, due toa lack of surveillance systems targeting adults or conducting genomic analysis. We conducted a genomic analysis of RSV strains circulating in Kyoto, Japan, using specimens obtained through regional surveillance.

**Methods:**

Between 2011 and 2018, 44 RSV-positive viral cultures were obtained from a public health laboratory in Kyoto. From 2021 to 2024, 344 RSV-positive clinical specimens were collected from 8 acute care hospitals and 1 testing center in Kyoto. These samples underwent whole-genome sequencing with viral nucleic acid target enrichment. Genomes were reconstructed using a mapping-based approach, and genomic clades as well as G gene-based clades (G-clades) were determined using Nextclade. Phylogenetic analysis was performed with 45 reference genomes representing each clade.

**Results:**

A total of 351 genome sequences (subtype A, n = 169; B, n = 182) with >90% coverage of the reference genomes were included in the analysis. Of these, 28% of RSV-A and 47% of RSV-B genomes were collected from adults. Among RSV-A genomes, clades A.3.1 and A.3.1.1 were common in the early 2010s. The A.D clade, which contains a duplication in the G gene, emerged in 2013, and its variants became dominant thereafter. The A.D.3 clade was the most prevalent after 2021, except in 2022, when A.D remained the most common. For RSV-B, clades B.3, B.D, and B.D.1 were detected in 2011. B.D.4.1.1 was predominant in 2021 and 2022, but was subsequently replaced by B.D.E.1 after 2023. Across both RSV subtypes, genomes from different age groups and facilities were closely related. While genomic clades corresponded to G-clades, they offered greater resolution, often dividing a single G-clade into multiple genomic clades.

**Conclusion:**

This study characterizes the regional genomic epidemiology of RSV in Kyoto, Japan, and reveals temporal shifts and transmission patterns. Our findings underscore the importance of continuous, high-resolution genomic surveillance to better understand RSV transmission and inform public health interventions.

**Disclosures:**

Yasufumi Matsumara, MD, PhD, Beckman Coulter: Research support for a collaborative project|Precision System Science: Research support for a collaborative project Miki Nagao, MD, PhD, Beckman Coulter: Research support for a collaborative project|Precision System Science: Research support for a collaborative project

